# Data-driven identification and classification of nonlinear aging patterns reveals the landscape of associations between DNA methylation and aging

**DOI:** 10.1186/s40246-023-00453-z

**Published:** 2023-02-11

**Authors:** Daigo Okada, Jian Hao Cheng, Cheng Zheng, Tatsuro Kumaki, Ryo Yamada

**Affiliations:** Center for Genomic Medicine, Graduate School of Medicine, Kyoto University, Kyoto, Japan

**Keywords:** Biomarker, Aging, DNA methylation, Epigenomics, Functional data analysis, Computational biology

## Abstract

**Background:**

Aging affects the incidence of diseases such as cancer and dementia, so the development of biomarkers for aging is an important research topic in medical science. While such biomarkers have been mainly identified based on the assumption of a linear relationship between phenotypic parameters, including molecular markers, and chronological age, numerous nonlinear changes between markers and aging have been identified. However, the overall landscape of the patterns in nonlinear changes that exist in aging is unknown.

**Result:**

We propose a novel computational method, Data-driven Identification and Classification of Nonlinear Aging Patterns (DICNAP), that is based on functional data analysis to identify biomarkers for aging and potential patterns of change during aging in a data-driven manner. We applied the proposed method to large-scale, public DNA methylation data to explore the potential patterns of age-related changes in methylation intensity. The results showed that not only linear, but also nonlinear changes in DNA methylation patterns exist. A monotonous demethylation pattern during aging, with its rate decreasing at around age 60, was identified as the candidate stable nonlinear pattern. We also analyzed the age-related changes in methylation variability. The results showed that the variability of methylation intensity tends to increase with age at age-associated sites. The representative variability pattern is a monotonically increasing pattern that accelerates after middle age.

**Conclusion:**

DICNAP was able to identify the potential patterns of the changes in the landscape of DNA methylation during aging. It contributes to an improvement in our theoretical understanding of the aging process.

**Supplementary Information:**

The online version contains supplementary material available at 10.1186/s40246-023-00453-z.

## Background

Aging affects the incidence of diseases such as cancer and dementia and is an important medical science research topic. The identification of biomarkers capable of measuring the degree of aging is a central theme in aging research. For example, the length of telomeres and the expression levels of the p16 gene are known to be correlated with chronological age and are representative biomarkers for aging [1, 2]. In recent years, considerable research has been conducted on identifying biomarkers using genomics data [3].

While changes in biomarkers for aging typically exhibit a linear relationship with chronological age, numerous nonlinear patterns in biomarker changes during aging have been reported. For example, the risk of age-related diseases does not increase proportionally with age, but rather, risk accelerates after middle age [4]. Human phenotype and disease risk also show different patterns of age-related changes [5], and nonlinear age-related changes in RNA and protein expression have also been reported [6–9]. For some DNA methylation sites, nonlinear changes in methylation intensity during aging following a power law have been reported [10]. While the patterns of aging are thought to reflect the underlying biological mechanisms, the overall landscape of nonlinear changes during aging is unknown.

Treating changes in the patterns of aging as a function of age allows for their mathematical analysis. Functional data analysis is a powerful statistical approach that can be applied to the analysis of the effect of age in a data-driven manner. Given that the functions are considered to be vectors with an infinite number of dimensions, numerous multivariate analysis methods have been extended to functional data. For example, functional principal component analysis can be used for dimension reduction of a functional dataset, and identification of principal patterns [11]. By developing a functional data analysis-based approach for large-scale genomics data, we can observe the overall landscape of age-related changes in biomarkers.

In this study, we propose a novel computational method based on nonlinear association analysis and functional data analysis to identify nonlinear biomarkers for aging and to clarify potential patterns of change during aging in a data-driven manner. We apply this approach to large-scale genome-wide DNA methylation data. Previous large-scale methylome analyses have revealed that some DNA methylation sites are hyper- or hypo- methylated during aging [12, 13]. In addition, DNA methylome information can be used as a powerful omics tool for developing biomarkers for aging at the epigenomic level. We show that age-related changes in the genome-wide DNA methylome typically have a linear pattern, but that nonlinear patterns are also present. We identified representative aging patterns of DNA methylation intensity and their variability. The findings presented here provide important insights into the link between DNA methylation and aging. Furthermore, the method developed in this study can be used to elucidate the landscape of patterns in aging-related changes and contribute to an improved theoretical understanding of the aging process.

## Results

### Novel computational workflow

**Fig. 1:**
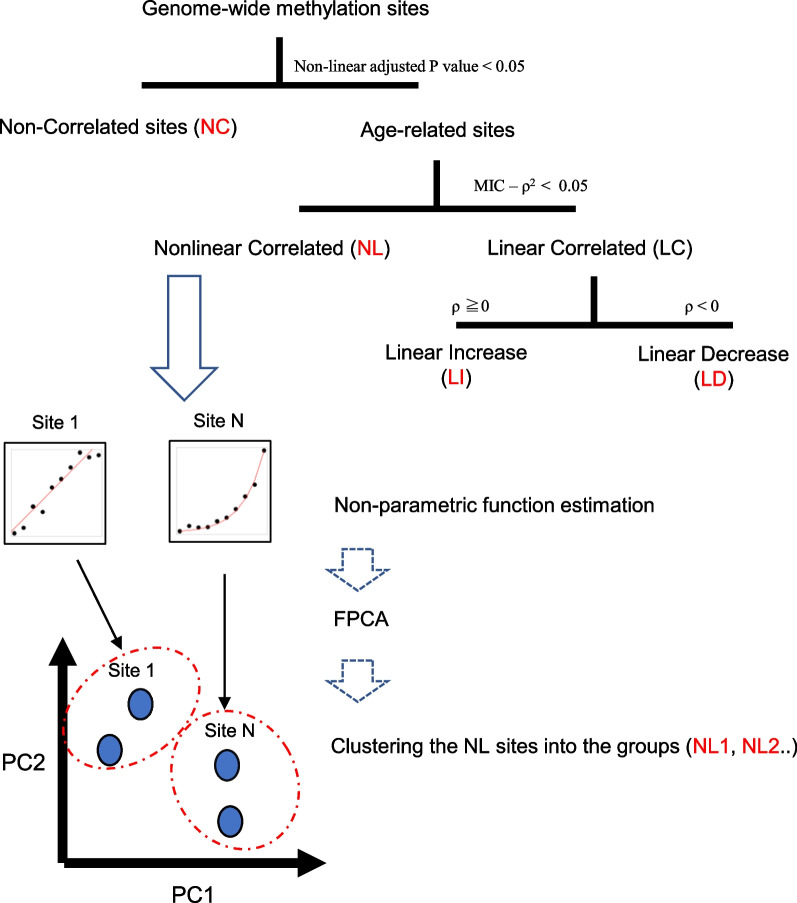
The outline of DICNAP method. Input data are a methylome dataset consisting of a matrix with subjects by DNA methylation site, and the elements of the matrix represent methylation intensities. Age information for the subjects is also required. After the multistep procedure, all methylation sites are classified as NC, LI, LD, NL1, NL2...NLK

To identify patterns of age-related change in a data-driven manner, we developed a novel computational approach which we refer to here as Data-driven Identification and Classification of Nonlinear Aging Patterns (DICNAP). Figure 1 shows a graphical abstract depicting our methodology. In addition to age information for the subjects, input data consist of the methylome dataset, which is a matrix with subjects by DNA methylation site, with the elements of the matrix representing methylation intensities. Briefly, the workflow consists of the following steps.

The first step involves performing a nonlinear correlation analysis to identify which of the methylation sites in the genome-wide DNA methylation site are associated with age. We used the maximal information coefficient (MIC), which is a nonlinear correlation index that ranges from zero to one, to capture any type of association between two variables [14]. We calculated the MIC between age and methylation intensity for each site, as well as the associated *P* value using permutation tests. Sites with Benjamini–Hochberg adjusted *P* values < 0.05 were considered to be age-associated sites for the downstream analysis [15]. All other sites were considered to be non-correlated sites (NC).

The second step is to classify the identified age-associated sites into linear and nonlinear patterns. MIC - $\rho ^2$ can be used as an index of nonlinearity, where $\rho$ is the Pearson correlation coefficient [14]. A larger MIC - $\rho ^2$ represents higher nonlinearity. We consider sites with a nonlinear index < 0.05 to be sites with a linear pattern. Among these linear sites, those with Pearson correlation coefficients $\ge$ 0 were considered to be linearly increasing (LI), and sites with Pearson correlation coefficients < 0 were considered to be linearly decreasing (LD). All other age-associated sites were defined as having a nonlinear (NL) pattern.

The third step is a nonparametric function estimation for the nonlinear age-associated methylation sites. To focus on the difference in the shape of the functions, we applied standard normalization of the methylation intensity for each site. We estimated the function for age-related changes by nonparametric regression with spline smoothing. As a result, the functional data for each site were generated as a scaled intensity on a grid separated by one year of age.

The fourth step is a functional principal component analysis (FPCA), which is used to embed the nonlinear age-associated methylation sites into a low-dimensional space based on the function patterns. FPCA takes a set of functional data as an input and gives principal component (PC) scores for each function. The eigenfunction shows the principal pattern that each PC represents. In this step, FPCA is applied to the age-related functions after the previous step and embeds the sites into a PC coordinate space. The PCs with a contribution rate > 1% were adopted as the top PCs for downstream analysis.

The final step comprises a multivariate analysis using PC coordinates for NL sites. Once the methylation sites are embedded into the PC coordinate space, ordinary multivariate analysis can be applied. Clustering of the PC coordinates allows for data-driven classification of DNA methylation sites with nonlinear patterns. We used K-means clustering and classified nonlinear sites into groups. These groups were named NL1, NL2...NLK, where K is the number of clusters automatically detected. For each of the K clusters, the median of the PC coordinate values was set to the center, and the typical pattern for each cluster was reproduced based on the PC coordinate values of the center, mean function and eigenfunctions.

Finally, our workflow classifies all methylation sites into NC, LI, LD, NL1, NL2...NLK, and the representative function pattern for NL sites. The details of the classification method are described in the Methods section. We applied the proposed method to a simulation dataset and describe the results of the simulation data analysis in Additional File 1: Section 1. Briefly, the simulation study showed that DICNAP can appropriately identify potential functional patterns and classify sites from an aging methylome dataset. However, a limitation of the method is that nonlinear changes that begin in very old age are difficult to detect.

### Genome-wide landscape of linear and nonlinear patterns for age-related change

**Fig. 2:**
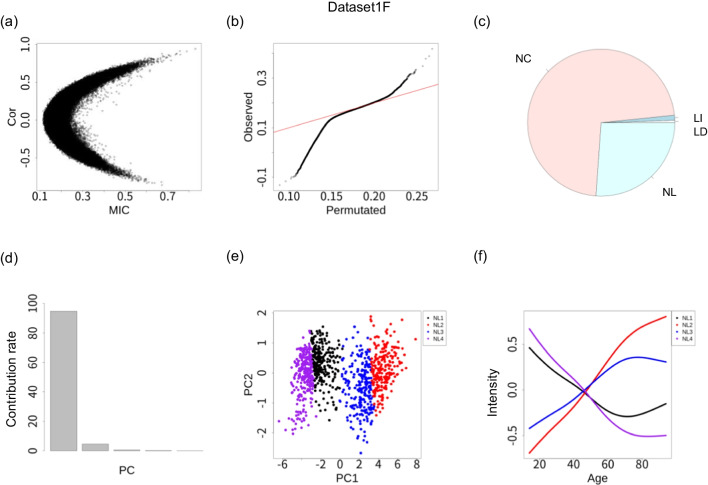
Analysis of methylation intensity function by age in Dataset1F. **a** Plots of MIC (X-axis) and Pearson’s correlation coefficient (Y-axis). Each dot represents a methylation site. **b** QQ plot of nonlinearity index. The nonlinearity index was defined as $MIC- \rho ^2$. The null distribution was generated by label permutation. The red line represents y = x. **c** Pie chart showing the genome-wide methylation sites for age-related association types: NC (non-correlated), LI (linear increase), LD (linear decrease), NL (nonlinear) . **d** Contribution rate for PC1–PC5. **e** PC1 and PC2 for 1000 randomly selected sites. The colors represent groups detected by clustering. **f** Representative functions for the identified NL groups (X-axis: age, Y-axis: scaled intensity)

We obtained a large-scale DNA methylome dataset of human blood from two previous aging studies from the NCBI Gene Expression Omnibus (GEO) database (GSE87571 and GSE40279) [12, 13]. The male and female datasets were analyzed separately because previous studies have reported that age-related changes in DNA methylation differ between men and women [16]. To eliminate SNPs and other effects, multimodal sites were excluded from the downstream analysis. Finally, from the GSE87571 dataset, the methylation intensity beta values for 479,461 sites from 388 female subjects between the ages of 14 and 94 were used to compile Dataset1F, and those for 479,634 sites from 341 male subjects between the ages of 15 and 87 were used to compile Dataset1M for downstream analysis. Similarly, from the GSE40279 dataset, the methylation intensity beta values for 454,171 sites from 338 female subjects between the ages of 21 and 101 were used to compile Dataset2F, and those for 454,852 sites from 318 male subjects between the ages of 19 and 96 were used to compile Dataset2M. Details of the dataset preprocessing steps are described in the Methods section. Among these four datasets, we used Dataset1F as the main dataset in our analysis, because it has the largest sample size (n=388). We used the three other datasets to evaluate the stability of the analysis. We applied DICNAP to all four datasets; the results for the main dataset (Dataset1F) are described in Fig. 2, and those for the other three datasets are described in Additional File 2.

Figure 2a shows a plot of the MICs and Pearson correlation coefficients. Sites with a high MIC tended to be plotted with a bias toward regions with high Pearson’s correlation coefficient, which suggests that the changes in the DNA methylation intensity during aging were dominated by monotonous changes. This finding, which is consistent with the high performance of previously reported biomarkers for aging based on linear relationships, was also found for the three other datasets.

Figure 2b shows a Quantile–Quantile plot (QQ plot) of the nonlinear index defined by $MIC - \rho ^2$. A null distribution was generated by subject label permutation. In this QQ plot, both inflation and deflation of the nonlinear index are observed. The inflation of the QQ plot shows that there are some methylation sites with nonlinear associations. The deflation of the QQ plot shows the existence of methylation sites with stronger linearity. These trends were also commonly observed in the other three datasets (Additional File 2), suggesting that some methylation sites have nonlinear monotonous intensity changes in addition to the linear changes. The breakdown of association types (NC, LI, LD, NL) in Dataset1F is shown in Fig. 2c. The percentage of age-associated sites (LI, LD, NL) was 27.7%, while those of Dataset1M, Dataset2F, and Dataset2M were 31.8%, 11.1%, and 4.1%, respectively. Although the same workflow and thresholds were applied to all of the datasets, differences in statistical power were observed across datasets.

To investigate biological phenomena associated with methylation sites that showed a linear response to age, we subjected LI and LD sites to Gene Ontology (GO) analysis. Additional File 3 shows all of the significant GO terms (false discovery rate (FDR) < 0.05). A total of 204 GO terms are significantly represented in the LI group, with the top five GO terms being “central nervous system neuron differentiation,” “cell fate commitment,” “DNA-binding transcription activator activity, RNA polymerase II-specific,” “pattern specification process,” and “synaptic membrane.” These GO terms were also significantly enriched in Dataset1M . In Dataset2F and Dataset2M, no GO terms were significantly enriched in the LI groups. In the LD group, no GO terms showed significant enrichment in any of the four datasets . The results for the other three datasets are shown in Additional File 4.

Figure 2d shows a bar plot of the contribution rate for PC1-PC5 in the main dataset. Most of the differences in function are explained by PC1 and PC2. Additional File 5 shows the mean function and eigenfunction for the FPCA for all four datasets. Except for the sign difference, their shapes are similar, although the mean function and $\psi _1(Age)$ behave differently, mainly at the edges. Figure 2e shows the results for the PC coordinate values with the identified group labels in the main dataset; four groups (NL1, NL2, NL3, and NL4) were identified as having nonlinear patterns. Indeed, in all datasets, the number of groups with nonlinear-type sites was four. Figure 2f shows the representative functions for the NL groups. While the representative functions were somewhat unstable among the four datasets, age-related hypomethylation patterns that moderated after around age 60 (NL1 and NL4 in Dataset1F) appeared as nonlinear patterns in all of the datasets (NL1 and NL3 in Dataset1M, NL2 in Dataset2F, and NL1 in Dataset2M).

We investigated the stability of the classification of NL sites by calculating the absolute Pearson correlation coefficients for the PC1, PC2, and PC3 values for the methylation sites classified as NL in all four datasets (Additional File 6). PC1 is more strongly correlated in the four datasets (Pearson’s correlation coefficient 0.94 - 0.98) than PC2 and PC3. Since the clustering of the NL group is explained mainly by PC1 (Fig. 2e), the classification of the methylation sites for NL patterns is considered to be stable across datasets.

We applied GO analysis to nonlinear site groups (NL1, NL2, NL3, and NL4). From the representative functions, NL1 and NL4 have nonlinearly decreasing patterns. In the NL1 group, the top five GO terms were “passive transmembrane transporter activity,” “regulation of membrane potential,” “extracellular matrix,” “transporter complex,” and “regulation of trans-synaptic signaling.” In the NL4 group, the top five GO terms were “regulation of cell morphogenesis,” “regulation of ion transmembrane transport,” “cell-cell junction,” “extracellular matrix structural constituent,” and “transmembrane receptor protein kinase activity.” All of these top GO terms were significantly enriched in one of the nonlinear decreasing patterns (NL1 or NL3) in Dataset1M. In contrast, in Dataset2F, only a total of five GO terms were significant for the nonlinear decreasing groups (either N1 or NL2) and contained no top GO terms in the main dataset. In Dataset2M, no GO terms were significant for the nonlinear decreasing groups (either N1 or NL2). The full results of the GO analysis using the main and other datasets are presented in Additional File 3 and Additional File 4, respectively.

In the main dataset, NL2 and NL3 are characterized by having nonlinearly increasing patterns. In NL2, the top five GO terms are “central nervous system neuron differentiation”, “cell fate commitment”, “appendage development”, “morphogenesis of a branching structure”, and “regulation of animal organ morphogenesis”. In NL3, the top five GO terms are “embryonic organ development,” “pattern specification process,” “cell fate commitment,” “synapse organization,” and “mesenchyme development.” These top GO terms were all also included in NL1 or NL3 of Dataset1M and are characterized by having increasing patterns. GO terms other than presynapse were also included in NL3 of Dataset2F and NL4 of Dataset2M, which are groups characterized by a nonlinear increasing pattern in each analysis.

We checked which groups contained known DNA methylation sites as biomarkers for aging in previous studies. For example, cg16867657 (annotated to *ELOVL2*), cg06639320 (annotated to *FHL2*), and cg16419235 (annotated to *PENK*) are known biomarkers for aging [17]. These sites showed positive linear correlations with age in the original study. In our analysis, these three sites were classified as LI in all four datasets.

In another study, cg02228185 (annotated to *ASPA*), cg25809905 (annotated to *ITGA2B*), and cg17861230 (annotated to *PDE4C*) were all shown to predict age by a linear model [18]. cg02228185 is grouped in LD in Dataset1F, Dataset1M, and Dataset2F. In Dataset2F, it is classified as NL1, which is a nonlinearly decreasing pattern. cg17861230 is grouped in LI in Dataset1F and Dataset1M, while it is grouped as NC in Dataset2F and NL4 in Dataset2M. cg25809905 is grouped in NL4 in Dataset1F; while it is LD in Dataset1M, NL1 in Dataset2F and NL1 in Dataset2M. In all three sites, the direction of increase and decrease is consistent with the original study. However, these sites did not always show linear associations, and in some cases, a nonlinear association was suggested.

We investigated which group the sites consisting Horvath’s epigenetic clock have been classified. Genome-wide DNA methylation-based biomarkers of aging are called epigenetic clocks. It has been demonstrated that the predicted age obtained using a linear model of the genome-wide methylation markers can predict chronological age [19]. In particular, Horvath’s epigenetic clock is a linear predictive equation consisting of 353 CpG sites and works with methylome data from a variety of human organs, suggesting a universal mechanism for why we age that transcends organ differences [20]. We therefore checked the associations among our identified groups and Horvath clock sites.

**Fig. 3:**
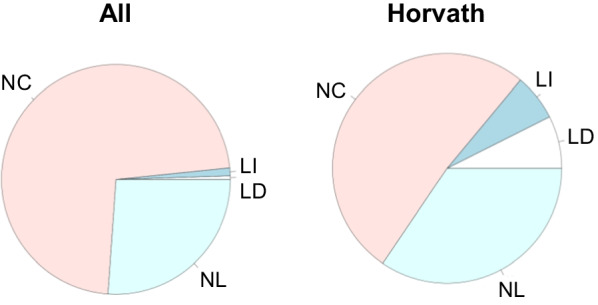
Pie charts for age-related correlation patterns of Horvath clock sites in Dataset1F. The left panel is a pie chart of all sites and is the same as in Fig. 2c. The right panel is a pie chart of the sites included in the Horvath clock. NC: non-correlated, LI: linear increase, LD: linear decrease, NL: nonlinear

Figure 3 shows a breakdown of association types (NC, LI, LD and NL) in the Horvath clock sites in Dataset1F. The percentage of age-associated sites among the Horvath clock sites was higher than that in the genome-wide methylation sites. While Horvath clock sites included a higher percentage of age-associated sites, they also included a meaningful percentage of NC and NL as well as LI and LD. This suggests that methylation sites in the Horvath clock include nonlinear changes and sites with only small or no stand-alone associations. The results for the other three datasets support this (Additional File 7).

### Applying the workflow to variability functions

Given that aging causes loss of control over biomolecular expression, the variability in biomarker expression should change during aging. When the variability in the DNA methylation intensity changes during aging, the residuals of the fitted regression function are expected to reflect this change. Previous studies have shown age-related increases in the variability of DNA methylation [10, 21–23]; however, it is not clear how this variability changes with age. We, therefore, applied the DICNAP to analyze the pattern of variability functions for methylation intensity.

**Fig. 4:**
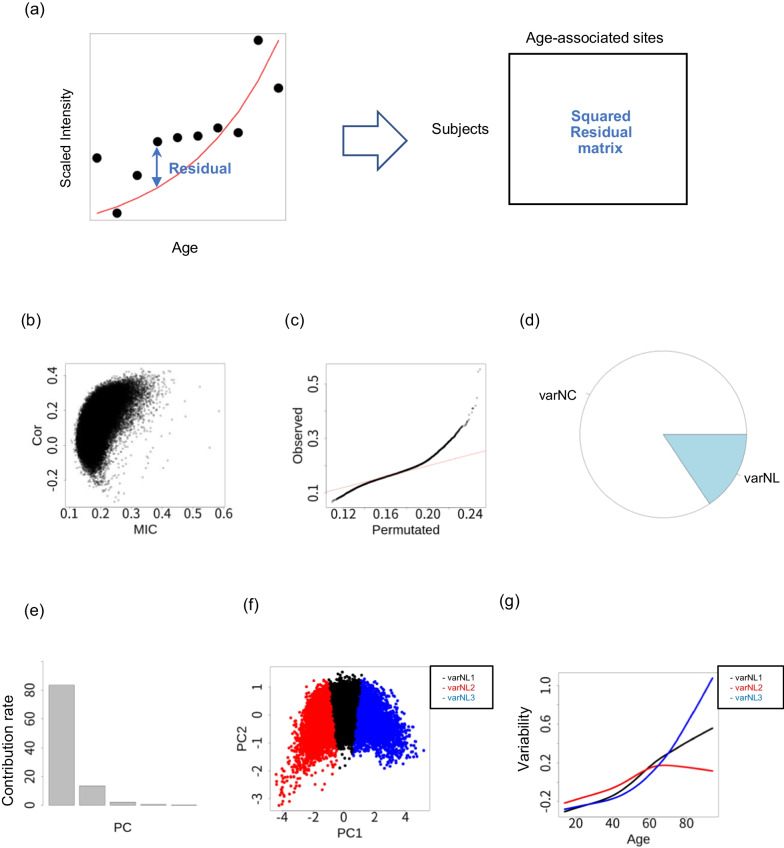
Analysis of variability function for age in Dataset1F. **a** Outline of the variability analysis using DICNAP. **b** Plots of MIC (X-axis) and Pearson’s correlation coefficient (Y-axis). Each dot represents a methylation site. **c** QQ plot of nonlinearity index, which is defined as $MIC- \rho ^2$. The null distribution was generated by label permutations. The red line represents y = x. **d** Pie charts for the genome-wide methylation sites of age-related correlation patterns: NC (non-correlated), LI (linear increase), LD (linear decrease), NL (nonlinear). **e** Contribution rate for PC1–PC5. **f** PC1 and PC2 in the FPCA analysis for 1000 randomly picked NL sites. The colors represent the groups detected by clustering. **g** Representative age-related functions for nonlinear sites (X-axis: age, Y-axis: scaled variability)

The DICNAP-based variability function analysis was applied as follows. The analysis was performed for age-associated sites (LI, LD, and NL). We applied nonparametric regression for all age-associated sites, computed the squares of the residuals, and created a squared residual matrix (Fig. 4a). The model for nonparametric regression is the same as with the intensity function analysis. We applied the DICNAP method to this matrix and classified the age-associated sites as varNC, varLI, varNL1, varNL2...varNLK in terms of variability. We conducted a simulation analysis and found that this approach effectively identified and classified the variability functions (Additional File 1: Section 2). In addition, the results also showed that linear variability changes tend to be identified as nonlinear, but these representative patterns can be identified as linear in downstream FPCA analyses.

Figure 4 shows the results of the variability function analysis for age-related sites in Dataset1F. Figure 4b shows a plot of the MICs and Pearson correlation coefficients. The plots are asymmetric and tend to have more sites with a positive correlation between variability and age. Figure 4c shows a QQ plot of the nonlinear index, suggesting the existence of nonlinear variability changes. This pattern was also identified in the other three datasets (Additional File 8).

After the classification of association types, no varLI and varLD sites were detected, and 15.6% of age-associated sites in Dataset1F had varNL characteristics (Fig. 4d). In Dataset1M, Dataset2F, and Dataset2M, 11.3%, 10.9%, and 15.4% of age-associated sites showed age-related variability changes, respectively. These findings suggested that about 10 $\sim$ 15% of age-associated sites stably show age-associated variability changes.

Subsequent FPCA analysis identified patterns of variability function in the methylome dataset. The shape of the mean function and eigenfunction was similar among all four datasets (Additional File 9). Figure 4e shows a bar plot of the contribution rate of principal coordinates in the main dataset. Figure 4f shows the results for PC coordinate values with clusters identified in the main dataset. Three groups (varNL1, varNL2, and varNL3) were identified as having a nonlinear variability pattern. Figure 4g shows the representative functions for the NL groups. As representatives, we identified patterns (varNL1 and varNL3) in which the variability increases monotonically and accelerates after middle age. This pattern was also identified in the other three datasets.

## Discussion

### Insights for aging biology

We developed a novel data-driven computational approach named DICNAP and applied it to four large-scale methylome datasets from two different studies. This method focuses on nonlinear changes in addition to linear changes, and we investigated the potential nonlinear patterns. One advantage of data-driven analysis is the ability to discover new biological knowledge from large datasets without using previous biological knowledge. Data-driven computational approaches also have been employed in genomics studies for analyzing a variety of datasets and tasks, and they have provided novel biological insights [24–26]. Our results provide the following new insights into the characteristics of DNA methylation and its effect on aging.

In this study, we found that age-related DNA methylation intensity is characterized by nonlinear monotonic demethylation, which slows down after around age 60. This trend is a stably observed pattern that was replicated among datasets. Errors in cytosine methylation during DNA replication are a passive cause of age-related DNA demethylation [27]. In addition, human cell division rates decrease with age [28]. The cause of this pattern may be that DNA demethylation is slowed by a decrease in the frequency of cell division with aging.

The results of our GO analysis clarified the biological processes associated with different aging patterns. In the nonlinear decreasing pattern, GO terms related to membrane transport were detected at the top of the list. In the linear or nonlinear increasing patterns, GO terms related to neurological or developmental processes were detected at the top of the list. However, no clear differences were found among each of the identified groups. These results suggest that nonlinear effects may be too weak to be linked to specific biological processes.

Our results suggest that the Horvath epigenetic clock is related to a mixture of sites with different association types. It has been reported that epigenetic clock sites are sensitive to the dataset being analyzed, and it is difficult to assess their functional relationship to the aging process [29]. It is possible that epigenetic clock sites include the methylation sites of nonlinear association types with small effects, which makes the interpretation of epigenetic clocks more difficult.

Nonlinear aging changes in the variability function were identified in approximately 10% of the sites whose methylation intensity changed with age. The identified stable pattern is a function of the variability that monotonically increases and accelerates after middle age. These sites suggest that the monotonically progressing breakdown of the control of the life system accelerates at middle age. Although active and passive processes are known to drive age-related changes in DNA methylation, this pattern may reflect the mechanisms that drive age-related changes.

### Limitations and future perspectives

One limitation of this study is related to the stability of the analysis due to the nonparametric approach. For example, the functional form of the edge region was unstable, and the detection power of age-associated sites differed across datasets. After carefully addressing these problems, we showed that some findings are highly stable. Although the biological insights that could be obtained from the data were limited, our results clarified the landscape of different patterns in aging-related changes and contribute to an improved theoretical understanding of the aging process.

Another limitation of this study is that cross-sectional data are used rather than longitudinal data. The functional patterns estimated in this study can be assumed to occur over the lifetime of an individual. On the other hand, since there are individual differences in the rate of aging, the use of cross-sectional data may be one reason why the results of this study are not so stable. Although it is difficult to obtain longitudinal methylome data spanning human ages from young to old, it would be meaningful to apply DICNAP to longitudinal omics data.

Interpreting the results of this study in terms of cellular subsets is an important issue for future study. The changes in bulk methylation levels are expressed as a mixed effect of changes in the proportion of cell subsets and cell subset-specific methylation changes [30]. A deconvolution approach using cellular subsets and based on methylome data would be effective for obtaining information on cell subset composition [31, 32]. In addition, recent studies have been employed cytometry and scRNA-seq approaches on a large scale; such findings allow us to directly obtain information on cellular heterogeneity [33–35]. Understanding of the biological mechanisms underlying the methylation biomarkers associated with aging would be greatly enhanced if the details of cell subset proportions and subset-specific nonlinear aging changes were known.

In recent years, attention has focused on the combined analysis of data from multiple omics layers, such as the epigenome, transcriptome, proteome, metabolome, and somatic genome [36]. DICNAP can be applied to a variety of aging omics data that are not limited to the methylome. An approach based on aging patterns across omics layers, including phenomics, is expected to provide a new direction for the construction of aging theory.

## Conclusion

In this study, we developed a novel computational method, called DICNAP, to identify biomarkers associated with aging and to clarify the potential patterns of changes that emerge during aging in a data-driven manner. By applying DICNAP to DNA methylome data, we clarified the landscape of associations between DNA methylation and aging and identified the representative pattern of age-related changes in methylation intensity and variability. DICNAP can be used to elucidate the landscape of different aging-related changes and to contribute to a theoretical understanding of the aging process.

## Methods

### DNA methylome dataset

We obtained the large-scale DNA methylome datasets from Illumina HumanMethylation450 BeadChips in previous aging studies from the NCBI GEO database(GSE87571 [12] and GSE40279 [13]). We renamed GSE87571 and GSE40279 as Dataset1 and Dataset2, respectively. These datasets were selected using the following procedure. First, we searched for “(GPL13534 [GEO Accession Number] ) AND aging” in the NCBI GEO database, sorted the output by sample size, and used the top two datasets as Dataset1 and Dataset2, respectively.

Dataset1 was obtained from the Northern Sweden Population Health Study, a health survey of the population, and includes data for residents aged 15 and older. We obtained the raw IDAT files from NCBI GEO. We applied SWAN normalization using the SWAN function and calculated the beta values as the methylation intensity from the IDAT files using the minfi package for R [37]. Using the diptest package for R, we tested for multimodality of the methylation intensity at each site [38]. Sites with multimodality (*P* value < 0.1) were removed from subsequent analyses. Three subjects whose age information was missing were also excluded from the analysis. We divided the dataset into male and female subjects, and these datasets were used for subsequent analyses.

Dataset2 was derived from two different cohorts, consisting of 426 Caucasian and 230 Hispanic individuals. We started the analysis from the preprocessed dataset downloaded from NCBI GEO. This dataset included only autosomal markers. The CpGs with a detection *P* value greater than 0.01 were considered missing; as a result, 830 markers with values greater than 5% were excluded in advance. This preprocessed dataset therefore contained a total of 473,034 markers. Sites exhibiting multimodality (i.e., *P*-value < 0.1) were removed from subsequent analyses. We divided the resulting data into male and female subjects, and these datasets were used for subsequent analyses.

### DICNAP method

In this section, we describe the technical aspects of our workflow. The first step involved a nonlinear correlation analysis to identify the methylation sites associated with age in a linear or nonlinear pattern from the genome-wide DNA methylation sites. We then calculated the MIC and R-squared between age and the methylation beta value for each site. To avoid numerical errors, the beta value was multiplied by $10^5$ before calculation. We also calculated the MIC and R-squared, once for each site, when the age label was permutated. The MIC was calculated using the minerva package for R . Using these procedures, we obtained the null distribution for MIC and $MIC - \rho ^2$. We then calculated the *P* values for nonlinear associations for each site based on the null distribution for the MIC. These *P* values were adjusted by the BH method, and the sites with adjusted *P* values < 0.05 were identified as age-related sites; other methylation sites were considered to be non-correlated sites (NC). Next, we classified the age-related methylation sites into linear and nonlinear patterns. We considered the sites with $MIC - \rho ^2$ < 0.05 to have a linear pattern. Among these linear sites, those with Pearson correlation coefficients $\ge$ 0 were considered to be linearly increasing (LI), and those with Pearson correlation coefficients < 0 were considered to be linearly decreasing (LD). All other age-related sites were defined as nonlinear patterns (NL). In the next step, we applied standard normalization to the methylation intensity for each site so that its mean = 0 and standard deviation = 1. We estimated the function for age-related changes using nonparametric regression with spline smoothing which is implemented by  the smooth.spline function in R with the settings all.knot = T and df = 4. The age grid was set to one-year intervals between the smallest and largest ages in the dataset. As a result, we obtained a set of functions and applied functional principal component analysis (FPCA) to the set of functions for nonlinear age-related methylation sites. We used the FPCA function in the fdapace package for R with the settings FVEthreshold = 1 and usedBinneddata = “OFF” [39]. As a result, we obtained the PC scores for each site, the mean function, and the eigenfunction. Based on the sum of the eigenvalues of all the calculated coordinates, the contribution rate was calculated. In the next step, we applied Kmeans clustering to the PC coordinates with a contribution rate > 1% as the top PCs. This implementation was conducted using the ClusterR package for R [40, 41]. The optimal number of clusters was detected automatically based on the distortion_fK criterion by the Optimal_Clusters_KMeans function, where the option setting was obtained from the vignette document (https://cran.r-project.org/web/packages/ClusterR/vignettes/the_clusterR_package.html). The maximum number of clusters whose distortion_fK criterion < threshold was adopted as the optimal number of clusters. Kmeans clustering was also conducted with KMeans_rcpp using this optimal number of clusters, where the option setting was also obtained from the vignette setting. Eventually, the NL sites were classified into the groups NL1, NL2...NLK, where K is the number of clusters that was automatically detected. For each of the K clusters, the median values of the PC coordinates were defined as the center point, and the representative pattern of each cluster *f*(*Age*) was reproduced by the formula $f(Age) = \mu (Age) + \sum ^M_{i=1} PC_i * \Psi _i(Age)$, where $\mu (Age)$ is the mean function, $PC_i$ is the coordinate value of the i-th principal component of the center point, $\Psi _i(Age)$ is the i-th eigenfunction, and M is the number of top PCs.

### Gene ontology analysis

Gene Ontology analysis for the group of methylation sites was conducted using the WebGestaltR package for R [42]. As databases, “geneontology_Biological_Process_noRedundant” “geneontology_Cellular_Component_noRedundant” and geneontology_Molecular_Function_noRedundant” were used. The FDR threshold was set to 0.05. All methylation sites after preprocessing for each dataset were used as the background methylation sites. The most significant GO terms were defined in the order of decreasing *P* value where, if there were multiple GO terms with the same *P* value, they were sorted in descending order by enrichment ratio.

## Supplementary information

**Additional file 1**: Description of simulation data for DICNAP analysis.

**Additional file 2**: Results of DICNAP analysis for Dataset1M, Dataset2F, and Dataset2M. The figure legend is the same as that of Fig. 2 in the main article.

**Additional file 3**: Full results of Gene Ontology analysis for Dataset1F. Each sheet is a WebgsealtR output for each methylation site group. Only significant GO terms (FDR < 0.05) are shown. Results for groups for which no significant GO terms were detected are omitted.

**Additional file 4**: Full results of Gene Ontology analysis for Dataset1M, Dataset2F, and Dataset2M.Each sheet is a WebgsealtR output for each methylation site group. Only significant GO terms (FDR < 0.05) are shown. Results for groups for which no significant GO terms were detected are omitted.

**Additional file 5**: Mean function and eigenfunction in the FPCA analysis for NL sites Ψ1, Ψ2, and Ψ3 are the eigenfunctions corresponding to PC1, PC2, and PC3, respectively. X-axis: age, Y-axis:scaled intensity.

**Additional file 6**: Analysis of PC coordinate stability among datasets table of absolute values of Pearson correlation coefficients for PC1, PC2, and PC3 values for NL sites among four datasets. This analysis was performed for sites classified as NL in all four datasets.

**Additional file 7**: Pie chart of groups in all four datasets.

**Additional file 8**: Results of DICNAP analysis for the variability function analysis in Dataset1M, Dataset2F, and Dataset2M. The figure legend of each panel is the same as that of Fig. 4 in the main article.

**Additional file 9**: Mean function and eigenfunction in the variability function analysis.Ψ1, Ψ2, and Ψ3 are the eigenfunctions corresponding to PC1, PC2, and PC3, respectively. X-axis: age, Y-axis:scaled variability.

## Data Availability

We uploaded a brief tutorial for our workflow using R using a Jupyter notebook at https://github.com/DaigoOkada/DICNAPtutorial.
