# Supplementary material for: Data-driven identification and classification of nonlinear aging patterns reveals the landscape of associations between DNA methylation and aging
Source: Hum Genomics. 2023 Feb 11;17:8. doi: 10.1186/s40246-023-00453-z (PMC9922449; doi:10.1186/s40246-023-00453-z)
Supplement: Supplementary file 1 — Additional file 1: Description of simulation data for DICNAP analysis. [file 40246_2023_453_MOESM1_ESM.pdf]

Description of simulation data analysis for DICNAP

Daigo Okada[1], Jian Hao Cheng[1], Cheng Zheng[1], Tatsuro Kumaki[1], and Ryo Yamada[1]

[1]Center for Genomic Medicine, Graduate School of Medicine, Kyoto University, Nanbusogo-Kenkyu-To-1, 5F, 53 Syogoin-Kawaramachi, Sakyo-ku, Kyoto 606-8507, Japan
*Corresponding author: Daigo Okada, dokada@genome.med.kyoto-u.ac.jp

1 Analysis of methylation intensity functions

We applied our proposed approach to a simulation dataset to confirm that it can appropriately identify potential function patterns and classify the methylation sites based on association types from a methylome dataset.

Data generation

Our simulation considered a total of nine methylation intensity function patterns of age: non-correlation (NC), linear increase (LI), linear decrease (LD), and six types of nonlinear pattern (NLI40: increase from age 40, NLD40: decrease from age 40, NLI60: increase from age 60, NLD60: decrease from age 60, NLI80: increase from age 80, and NLD80: decrease from age 80). The formula of each pattern is represented as follows, and Fig. 1 shows each function.

$$f_{NC}(Age) = 0.5$$
$$f_{LI}(Age) = 0.01 + 0.01 * Age$$
$$f_{LD}(Age) = 0.99 - 0.01 * Age$$
$$f_{NLI40}(Age) = \begin{cases} 0.1 & \text{if } Age < 40, \\ 0.1 + 0.01 * (Age - 40) & \text{if } Age \geq 40, \end{cases}$$
$$f_{NLD40}(Age) = \begin{cases} 0.9 & \text{if } Age < 40, \\ 0.9 - 0.01 * (Age - 40) & \text{if } Age \geq 40, \end{cases}$$
$$f_{NLI60}(Age) = \begin{cases} 0.1 & \text{if } Age < 60, \\ 0.1 + 0.01 * (Age - 60) & \text{if } Age \geq 60, \end{cases}$$
$$f_{NLD60}(Age) = \begin{cases} 0.9 & \text{if } Age < 60, \\ 0.9 - 0.01 * (Age - 60) & \text{if } Age \geq 60, \end{cases}$$
$$f_{NLI80}(Age) = \begin{cases} 0.1 & \text{if } Age < 60, \\ 0.1 + 0.01 * (Age - 80) & \text{if } Age \geq 60, \end{cases}$$
$$f_{NLD80}(Age) = \begin{cases} 0.9 & \text{if } Age < 60, \\ 0.9 - 0.01 * (Age - 80) & \text{if } Age \geq 60, \end{cases}$$

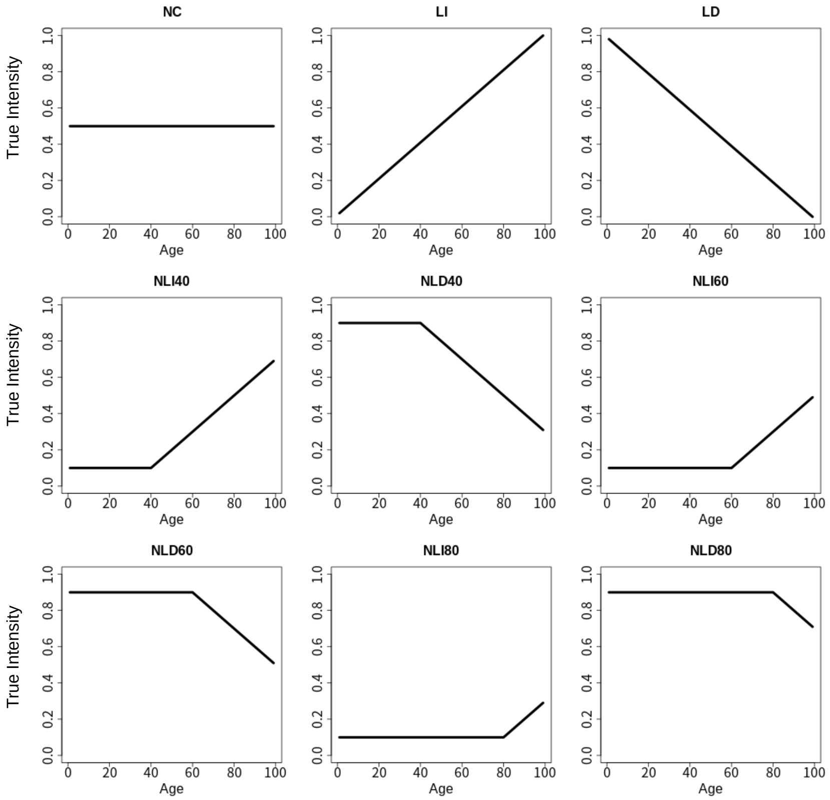

Figure 1: True intensity functions for the nine groups.

Based on these methylation intensity functions, we generated methylation data for 300 people $\times$ 2800 sites. The age of people was randomly determined to be between 1 and 100 years old. The 2800 methylation sites consisted of 1200 NC and 200 each of LI, LD, NLI40, NLD40, NLI60, NLD60, NLI80, and NLD80. The methylation intensity value of i-th person's j-th site, $(m_{i,j})$, was generated as follows.

$$
\begin{aligned}
&\mu_{i,j} = f^j(Age_i) \\
&a_{i,j} = \frac{\mu_{i,j} * b}{1 - \mu_{i,j}} \\
&b = 1 \\
&m_{i,j} \sim Beta(a = a_{i,j}, b = 1)
\end{aligned}
$$

where $f^j(Age)$ is the function of the j-th methylation site, and Age_i is the i-th person's age. The methylation intensity value was sampled from the Beta distribution, with the mean value being the function value of each person's age. As a result, an artificial methylation intensity of 300 people of various ages by 2800 sites consisting of nine types of age-related pattern (NC, LI, LD, NLI40, NLD40, NLI60, NLD60, NLI80, and NLD80) was obtained. An example of the simulated methylation intensity in each group is drawn in Fig. 2.

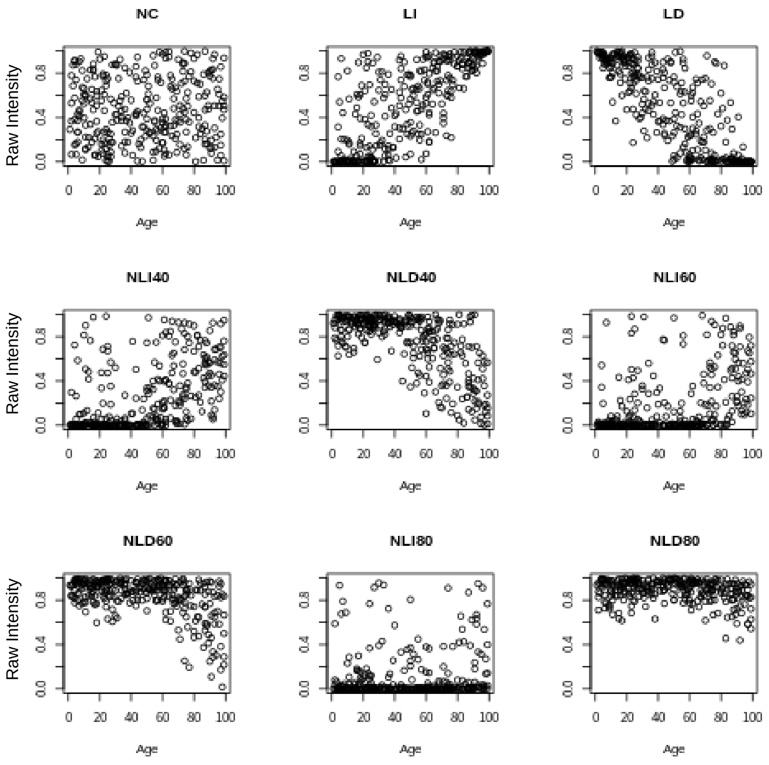

Figure 2: Example data for methylation sites for each group.

Result

We applied DICNAP to this simulated data and examined its performance for the identification and classification of DNA methylation sites based on their function pattern.

The nonlinear index ($MIC - \rho^2$) for each true group (Fig. 3(a)) shows that this index can work to classify linear and nonlinear patterns. Fig. 3(b) is the PC coordinate plot of FPCA with a true label, which suggests that FPCA can identify the differences in function patterns. Fig. 3(c) shows the FPCA results (bar plot of the contribution rate, mean, and eigen functions).

Fig. 3(d) shows the confusion matrix of the true and estimated groups, the PC plots with NL groups, and representative functions of each NL group. It shows that the estimated NL group strongly corresponds to the true group. The estimated NC, LI, and LD groups contained most of the true NC, LI, and LD sites, respectively. Moreover, the estimated NL sites corresponded as follows: NL1-NLI40, NL2-NLD60, NL3-NLI60, NL4-NLI80, NL5-NLD40, and NL6-NLD80, although NL4 was a mixed group of a part of NLI80 and misclassified NC. Our approach identified all six potential nonlinear groups. The estimated representative function for each group is consistent with these true functional forms. These results suggest that our approach can identify and classify both linear and nonlinear changes in a data-driven manner among aging methylome datasets. However, most of NLD80 and NLI80 sites were classified as NC. The representative functions of NL4 (corresponding to NLI80) and NL6 (corresponding to NLD80) showed changes prior to age 80. These results suggest that changes occurring from very old age are difficult to capture.

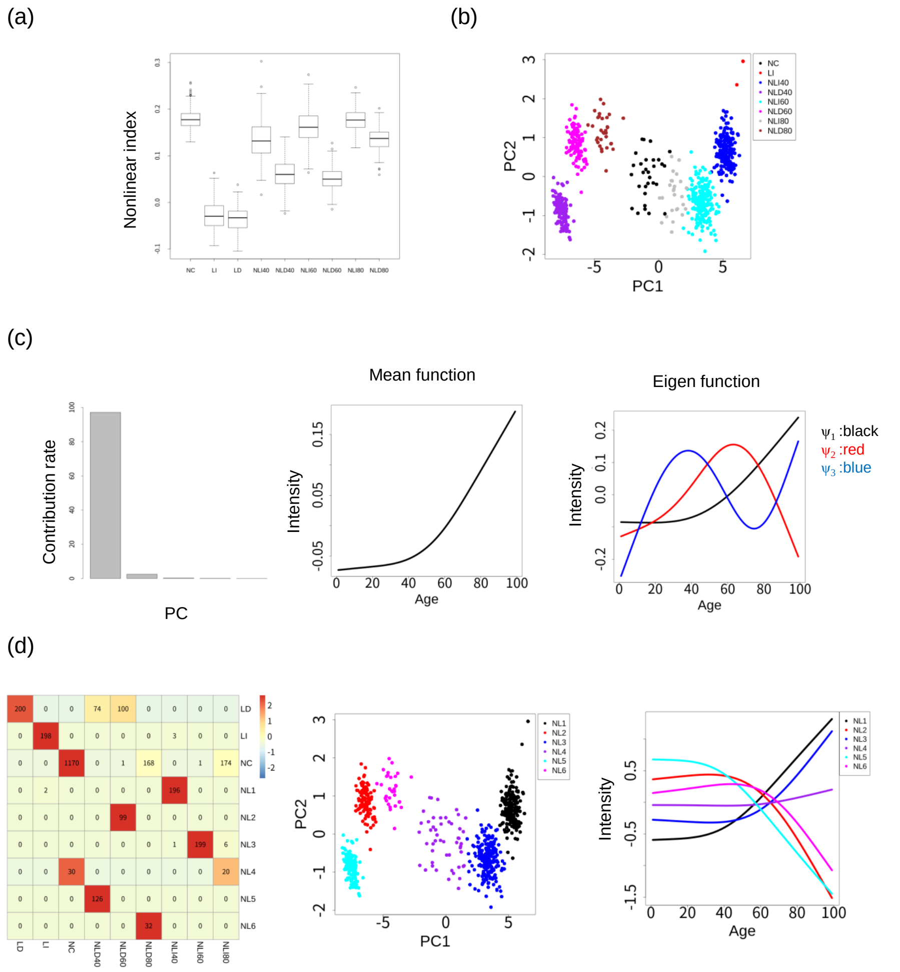

Figure 3: (a) Boxplot of nonlinear index defined as $MIC - \rho^2$ for each true intensity function group. (b) PC1 and PC2 of FPCA analysis with true labels. (c) Contribution rate, mean function, and eigen function of FPCA analysis. (d) Left panel: confusion matrix between nine true function groups (NC, LI, LD, NLD40, NLD60, NLD80, NLI40, NLI60, and NLI80) and the estimated groups (NC, LI, LD, NL1-NL6). The element of the matrix represents the number of methylation sites. The color represents the row-wise standard normalized number of sites. The estimated groups show high purity for a particular true function group. The PC plots with the estimated NL methylation groups (middle panel) and the representative function for each NL group (right panel) are shown.

2 Analysis for the variability functions

We applied this proposed method to a simulation dataset to confirm that this workflow can appropriately identify differences in variability function pattern.

Data generation

For the validation of DICNAP-based variability function analysis, we applied the proposed method to simulated data. We simulated a situation in which three different patterns of variability depending on age were added to the base linearly increasing intensity function. The methylation intensity function is common for all sites as follows.

$$f(Age) = 0.4 + 0.002 * Age$$

We considered three patterns of age-related variability change: non-correlated (NC), linear (L), and nonlinear (NL). The variability functions of age for each pattern were set as follows.

$$\begin{aligned}
&g_{NC}(Age) = 0.1 \\
&g_{L}(Age) = 0.05 + 0.002 * Age \\
&g_{NL}(Age) = \begin{cases} 0.1 & \text{if } Age < 40, \\ 0.1 + 0.002 * (Age - 40) & \text{if } Age \geq 40, \end{cases}
\end{aligned}$$

We generated methylation data for 300 people × 600 sites. The age of people was randomly determined to be between 1 and 100 years old. The 600 methylation sites consisted of 200 each of NC, L, and NL according to the variability pattern. The methylation intensity value of i-th person's j-th site, $(m_{i,j})$, was generated as follows.

$$\begin{aligned}
&\mu_{i,j} = f(Age_i) \\
&e_{i,j} \sim Normal(0, g^j(Age_i)) \\
&m_{i,j} = \begin{cases} 0 & \text{if } \mu_{i,j} + e_{i,j} < 0, \\ \mu_{i,j} + e_{i,j} & \text{if } 0 < \mu_{i,j} + e_{i,j} < 1, \\ 1 & \text{if } \mu_{i,j} + e_{i,j} > 1. \end{cases}
\end{aligned}$$

where $g^j(Age)$ is the function of the j-th methylation site, and Age_i is the i-th person's age. As a result, an artificial methylation intensity of 300 people of various ages with 600 sites consisting of three types of age-related variability

pattern (varNC, varL, and varNL) was obtained. An example of the simulated methylation intensity in each group is drawn in Fig. S4(b). We applied DICNAP for the variability function analysis to this simulated data and evaluated the performance.

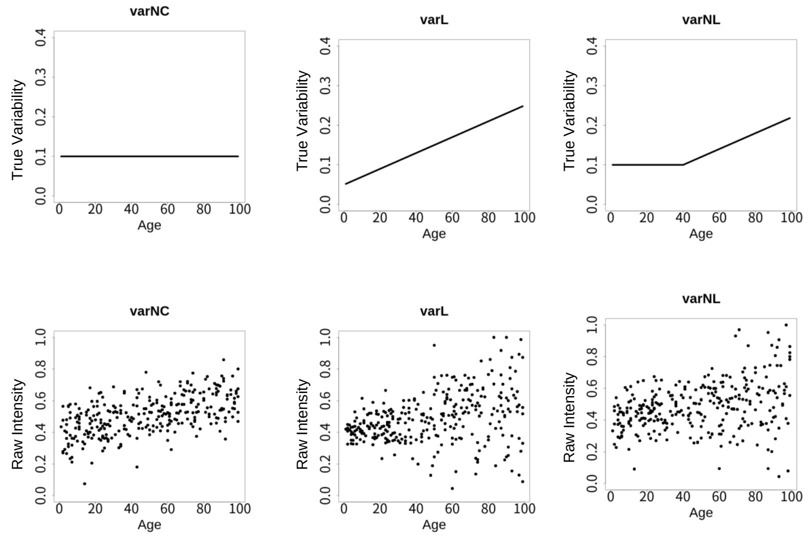

Figure 4: True intensity functions and the example of simulated site for the three groups.

Result

Fig. 5(a) shows that FPCA can identify the differences on the PC plot. Fig. 5(b) shows the mean and eigen functions of FPCA, and Fig. 5(c) shows the confusion matrix true label (varNC, varL, and varNL) and estimated groups. No sites were identified as LI or LD. A total of five NL sites (NL1-NL5) were identified according to variability functions, although redundancy exits (Fig. 5(c)). NL1 and NL4 correspond to true label varNL, NL3 and NL5 correspond to misclassified varL, and NL2 corresponds to a misclassified varNC. In fact, the representative functions of NL3 and NL5 are drawn as linear functions. Although the true linear functions were identified as varNL, the representative function was identified as an almost linear line. NL1 and NL4 are drawn as nonlinear functions increasing at about age 40, which corresponds to the true nonlinear variability function. These results suggest that our approach can successfully identify the groups corresponding to the potential variability pattern, although there is some redundancy and linear sites tended to be identified as NL

sites. It can also identify the potential variability function shape in a data-driven manner.

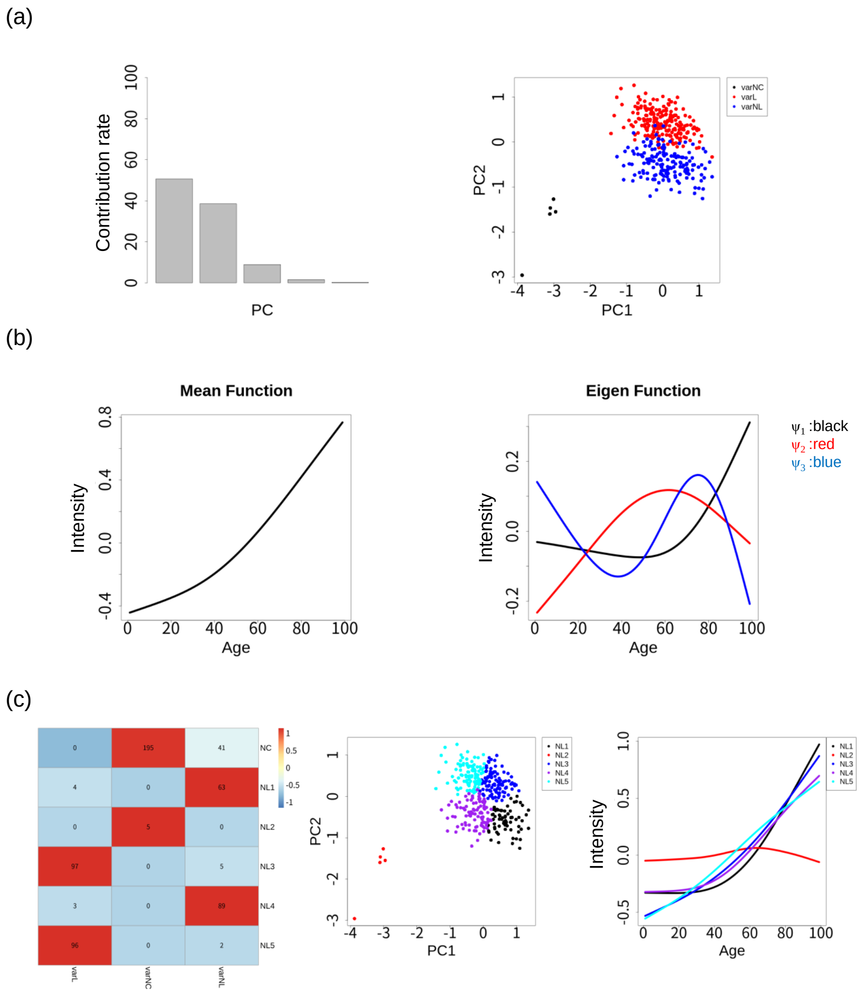

Figure 5: (a) Contribution rate of PC coordinates and PC coordinate plot of the estimated NL sites with a true label. (b) Mean function and eigen function for PC1-3. (d) Left panel: confusion matrix between three true function groups (varNC, varL, and varNL) and the estimated groups (NC, NL1-NL5). The element of the matrix represents the number of methylation sites. The color represents the row-wise standard normalized number of the sites. Middle panel: PC coordinate plots with identified NL groups. Right panel: representative functions for each NL group. The function shape is consistent with the true function assigned from the confusion matrix.

Conclusion

This simulation study has shown that DICNAP can appropriately identify potential function patterns and classify sites from aging methylome datasets. This can work for both methylation intensity function and variability functions. In addition, as a limitation of the method, the simulation analysis suggested that nonlinear changes that begin in very old age is difficult to detect. In addition, in the variability function analysis, liner variability change tend to be identified as nonlinear but these representative pattern can be identified as linear line in downstream FPCA analysis.
