# Supplementary figures and images for: Data-driven identification and classification of nonlinear aging patterns reveals the landscape of associations between DNA methylation and aging
Source: Hum Genomics. 2023 Feb 11;17:8. doi: 10.1186/s40246-023-00453-z (PMC9922449; doi:10.1186/s40246-023-00453-z)

# Dataset1M

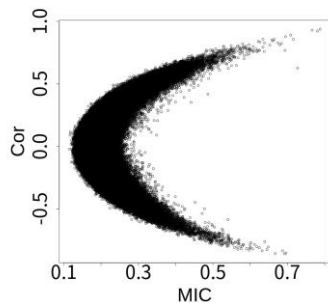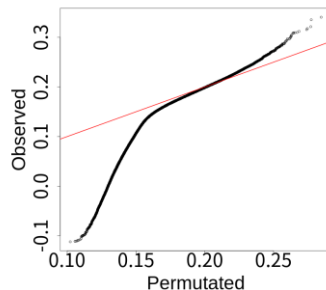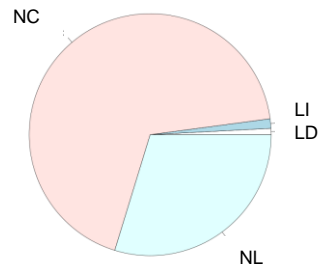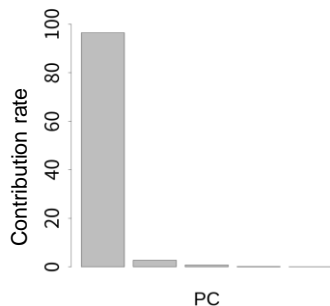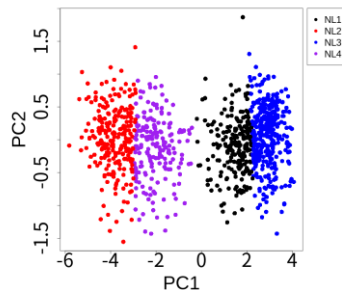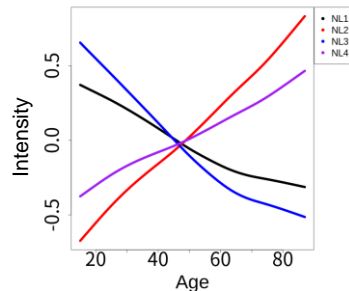

# Dataset2F

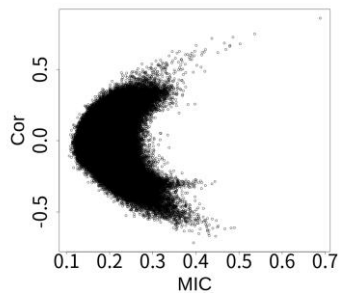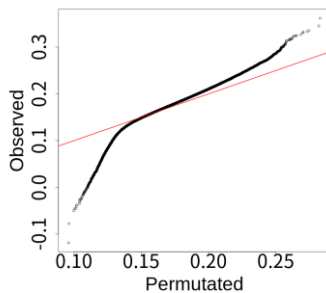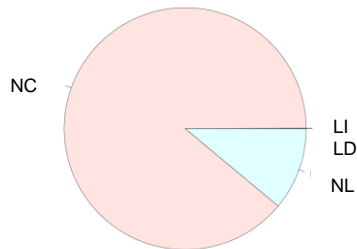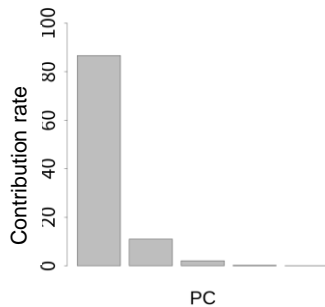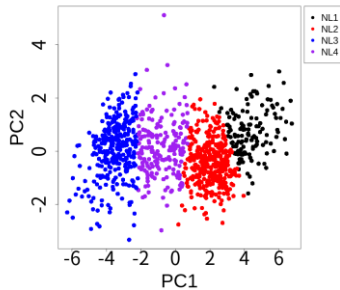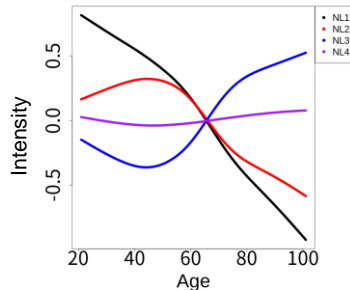

# Dataset2M

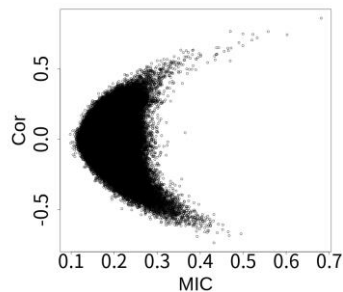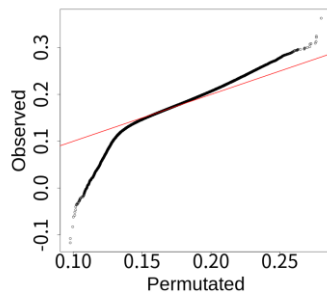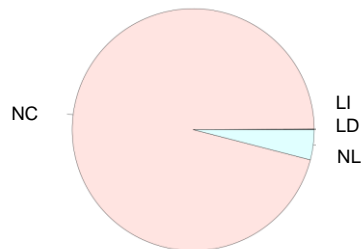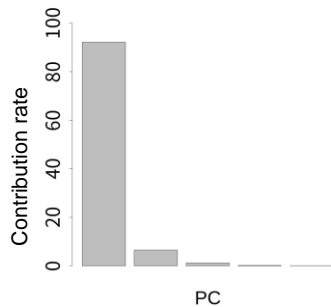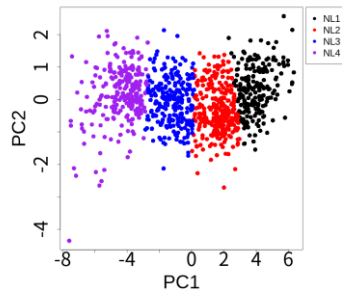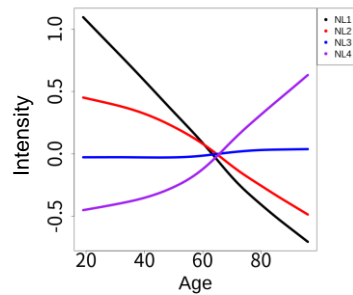

Supplement: Supplementary file 2 — Additional file 2: Results of DICNAP analysis for Dataset1M, Dataset2F, and Dataset2M. The figure legend is the same as that of Fig. 2 in the main article. [file 40246_2023_453_MOESM2_ESM.pdf]

# Mean Function

# Eigen Function

Dataset1F

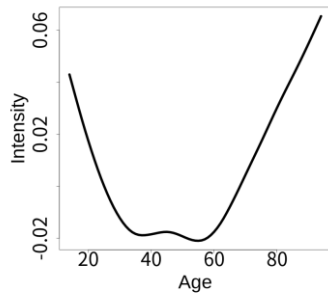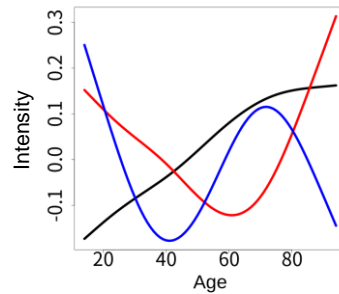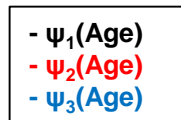

Dataset1M

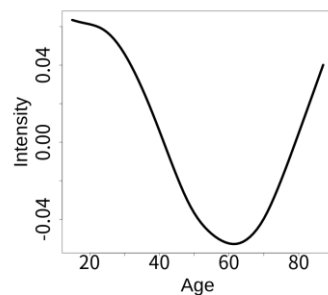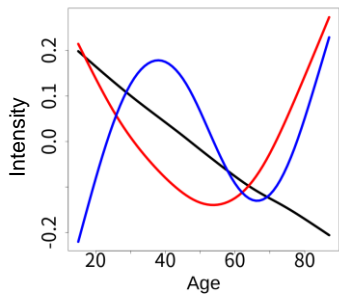

Dataset2F

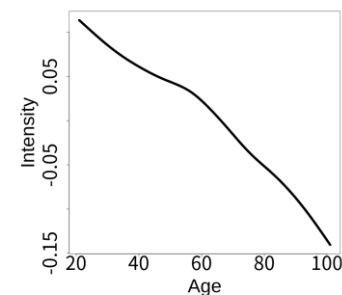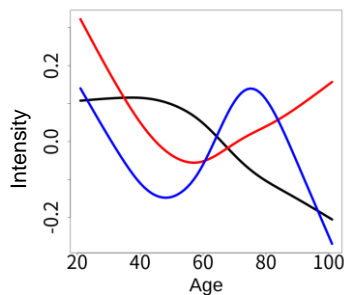

Dataset2M

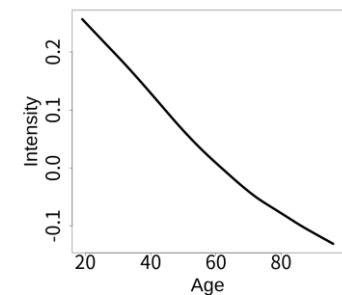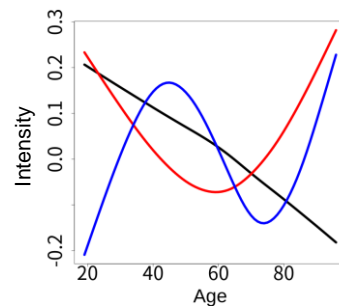

Supplement: Supplementary file 5 — Additional file 5: Mean function and eigenfunction in the FPCA analysis for NL sites Ψ1, Ψ2, and Ψ3 are the eigenfunctions corresponding to PC1, PC2, and PC3, respectively. X-axis: age, Y-axis:scaled intensity. [file 40246_2023_453_MOESM5_ESM.pdf]

## All

## Horvath

**Dataset1F**

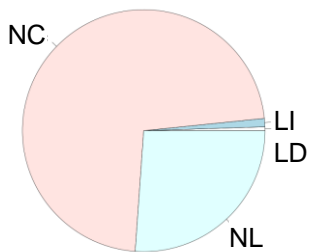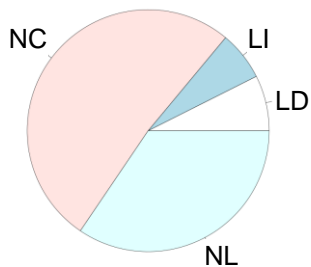

**Dataset1M**

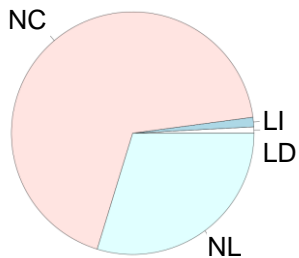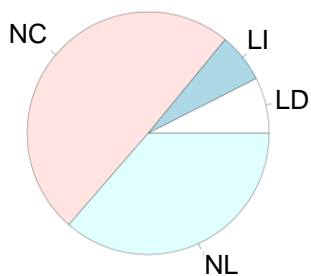

**Dataset2F**

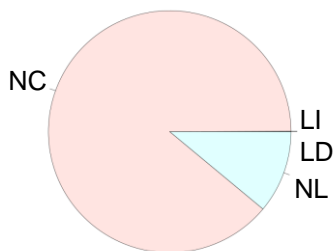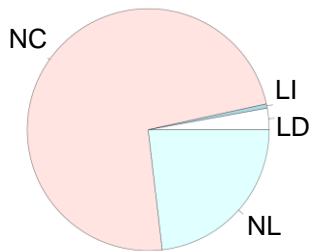

**Dataset2M**

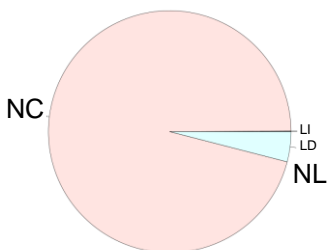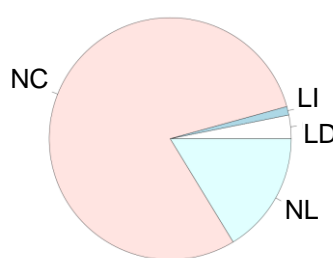

Supplement: Supplementary file 7 — Additional file 7: Pie chart of groups in all four datasets. [file 40246_2023_453_MOESM7_ESM.pdf]

## Dataset1M

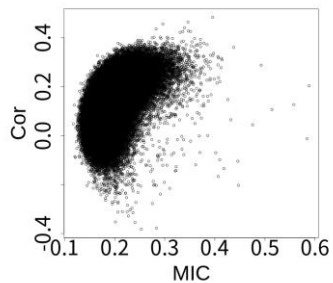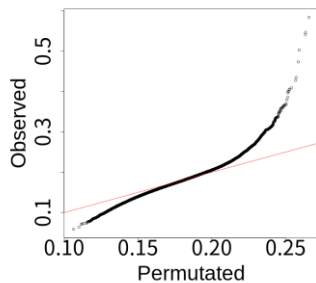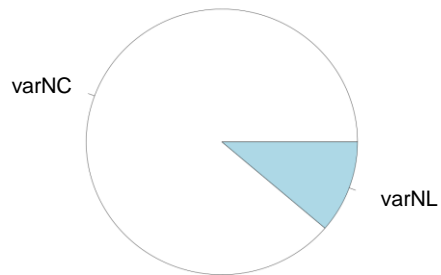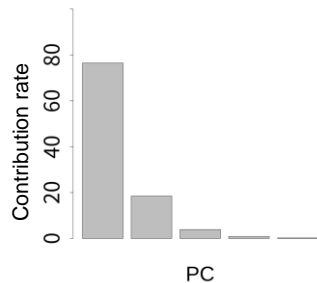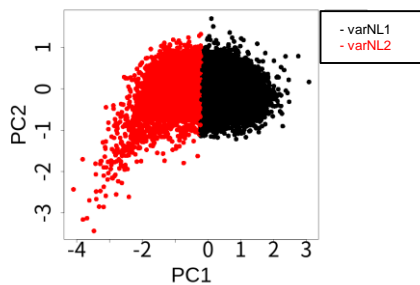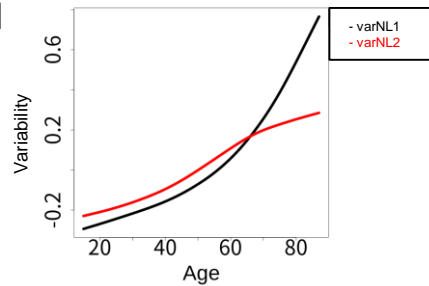

## Dataset2F

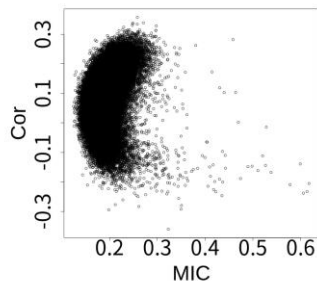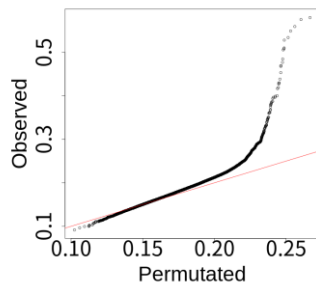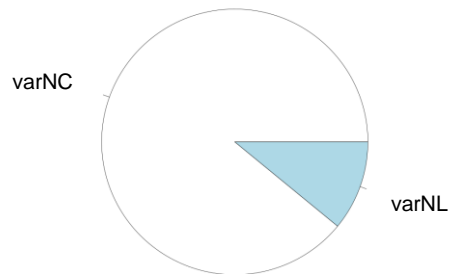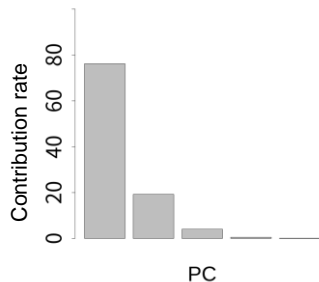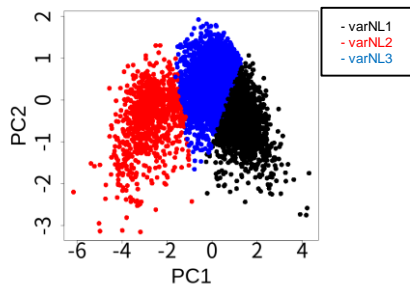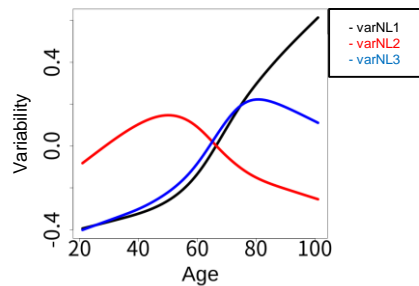

## Dataset2M

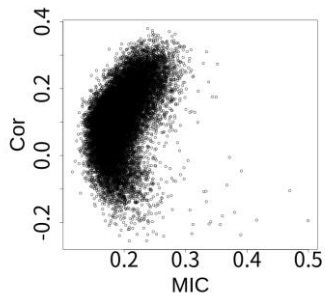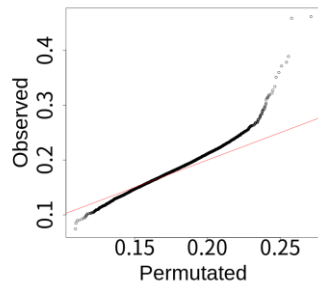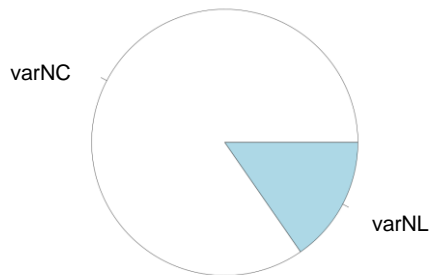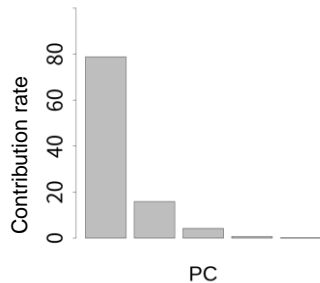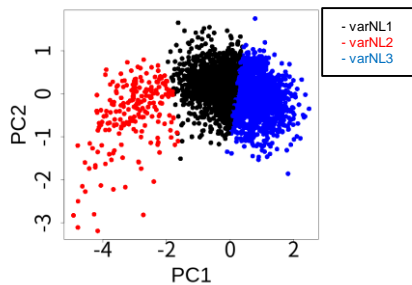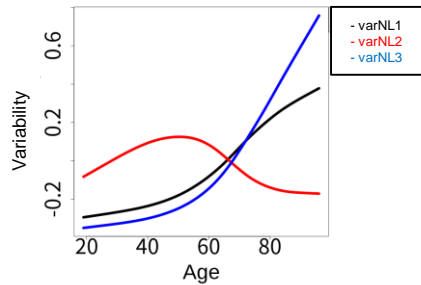

Supplement: Supplementary file 8 — Additional file 8: Results of DICNAP analysis for the variability function analysis in Dataset1M, Dataset2F, and Dataset2M. The figure legend of each panel is the same as that of Fig. 4 in the main article. [file 40246_2023_453_MOESM8_ESM.pdf]
