# Supplementary material for: Data-driven identification and classification of nonlinear aging patterns reveals the landscape of associations between DNA methylation and aging
Source: Hum Genomics. 2023 Feb 11;17:8. doi: 10.1186/s40246-023-00453-z (PMC9922449; doi:10.1186/s40246-023-00453-z)
Supplement: Supplementary file 6 — Additional file 6: Analysis of PC coordinate stability among datasets table of absolute values of Pearson correlation coefficients for PC1, PC2, and PC3 values for NL sites among four datasets. This analysis was performed for sites classified as NL in all four datasets. [file 40246_2023_453_MOESM6_ESM.pdf]

PC1

	1F	2F	1M	2M
1F	1	0.94	0.98	0.95
2F	0.94	1	0.94	0.97
1M	0.98	0.94	1	0.95
2M	0.95	0.97	0.95	1

PC2

	1F	2F	1M	2M
1F	1	0.26	0.55	0.05
2F	0.26	1	0.11	0.1
1M	0.55	0.11	1	0.29
2M	0.05	0.1	0.29	1

PC3

	1F	2F	1M	2M
1F	1	0.06	0.08	0.19
2F	0.06	1	0.01	0.02
1M	0.08	0.01	1	0.08
2M	0.19	0.02	0.08	1
