# Supplementary material for: Data-driven identification and classification of nonlinear aging patterns reveals the landscape of associations between DNA methylation and aging
Source: Hum Genomics. 2023 Feb 11;17:8. doi: 10.1186/s40246-023-00453-z (PMC9922449; doi:10.1186/s40246-023-00453-z)
Supplement: Supplementary file 9 — Additional file 9: Mean function and eigenfunction in the variability function analysis.Ψ1, Ψ2, and Ψ3 are the eigenfunctions corresponding to PC1, PC2, and PC3, respectively. X-axis: age, Y-axis:scaled variability. [file 40246_2023_453_MOESM9_ESM.pdf]

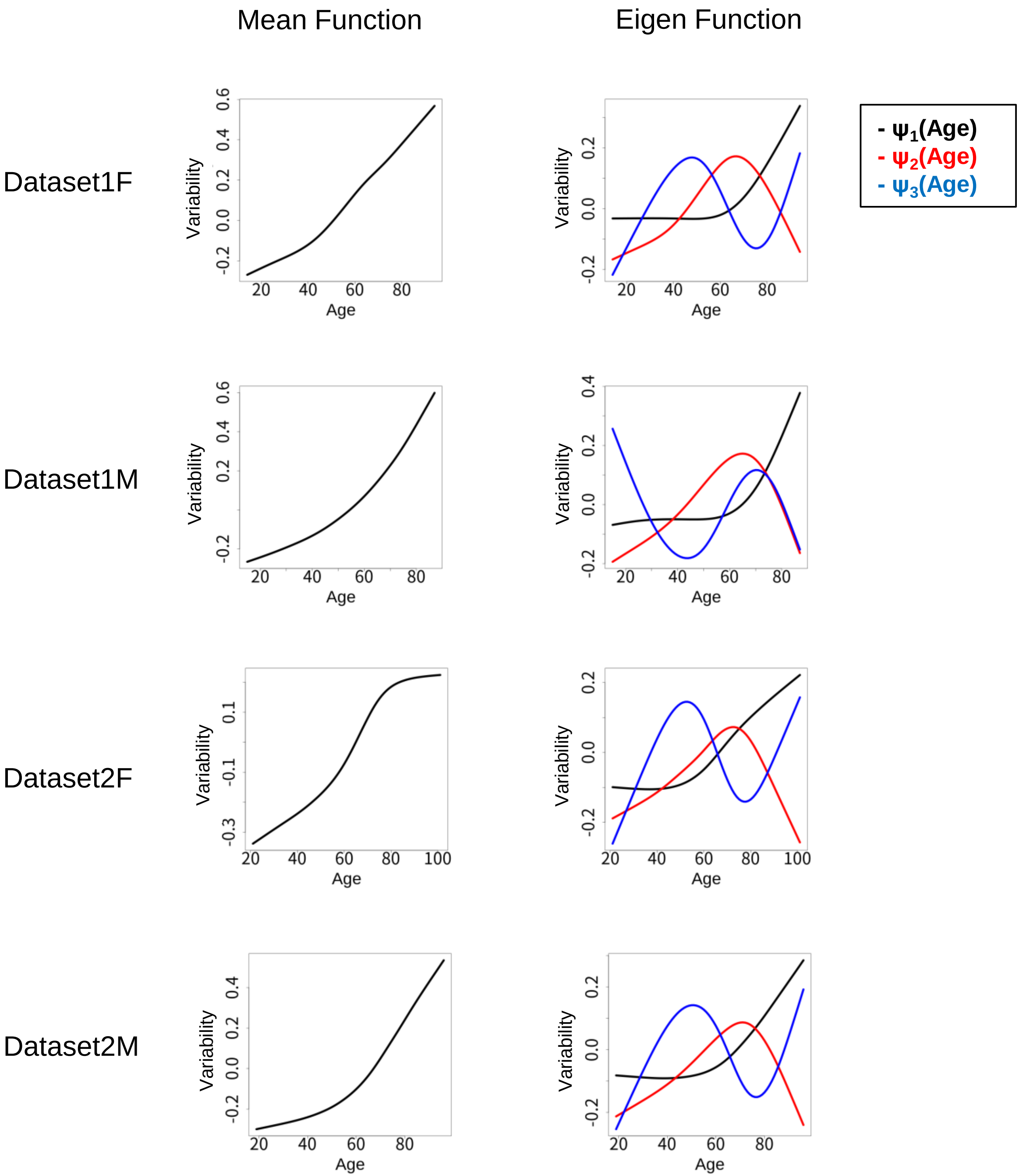

Mean Function
Eigen Function
Dataset1F
Dataset1M
Dataset2F
Dataset2M
Variability
Age
- ψ1(Age)
- ψ2(Age)
- ψ3(Age)
